# Divergence in the transcriptional landscape between low temperature and freeze shock in cultivated grapevine (*Vitis vinifera*)

**DOI:** 10.1038/s41438-018-0020-7

**Published:** 2018-03-01

**Authors:** Jason P. Londo, Alisson P. Kovaleski, Jacquelyn A. Lillis

**Affiliations:** 1 United States Department of Agriculture, Agricultural Research Service, Grape Genetics Research Unit, 630 W. North Street, Geneva, NY USA; 2 School of Integrative Plant Science, Horticulture section, Cornell University-New York State Agricultural Experiment Station, 630 W. North Street, Geneva, NY USA; 3 Genomics Research Center, University of Rochester Medical Center, Rochester, NY USA

## Abstract

Low-temperature stresses limit the sustainability and productivity of grapevines when early spring frosts damage young grapevine leaves. Spring conditions often expose grapevines to low, but not damaging, chilling temperatures and these temperatures have been shown to increase freeze resistance in other model systems. In this study, we examined whole-transcriptome gene expression patterns of young leaf tissue from cuttings of five different grapevine cultivars, exposed to chill and freeze shock, in order to understand the underlying transcriptional landscape associated with cold stress response. No visible damage was observed when grapevine leaves were exposed to chilling temperatures while freeze temperatures resulted in variable damage in all cultivars. Significant differences in gene expression were observed between warm control conditions and all types of cold stress. Exposure to chill stress (4 °C) versus freezing stress (−3 °C) resulted in very different patterns of gene expression and enriched pathway responses. Genes from the ethylene signaling, ABA signaling, the AP2/ERF, WRKY, and NAC transcription factor families, and starch/sucrose/galactose pathways were among the most commonly observed to be differentially regulated. Preconditioning leaves to chill temperatures prior to freezing temperatures resulted in slight buffering of gene expression responses, suggesting that differences between chill and freeze shock perception complicates identification of candidate genes for cold resistance in grapevine. Overall, the transcriptional landscape contrasts observed between low temperature and freezing stresses demonstrate very different activation of candidate pathways impacting grapevine cold response.

## Plant stress responses: How cold is too cold for grapevines?

An exploration of gene activity in grapevine (*Vitis vinifera*) suggests that these plants respond very differently to cold versus freezing temperatures. Low temperatures during the growing season (such as late spring frosts) can cause great damage to developing leaves of crops such as grapevines, particularly when grown outside their natural climatic zone. Jason Londo, of the US Department of Agriculture and Cornell University, and colleagues, explored the molecular response of young grapevine leaves to both cold (4 °C) and freezing (−3 °C) temperatures. They found very different responses in terms of genes and metabolic pathways that were activated or deactivated under the two regimes. In contrast to other species, exposure to cold did not predispose the vines to resist freezing temperatures. The research may help to identify potential targets for genetic manipulation to produce frost-resistant grapevine varieties.

## Introduction

Environmental stresses are the leading factors limiting the current and future expansion of grapevine production in the United States. As a Mediterranean-adapted species, cultivated grapevine (*Vitis vinifera*) is best suited to climates with mild winter conditions and with relatively consistent temperature change in autumn and spring. However, grapevine is often grown outside of this climatic niche in regions that have severe winter conditions, restricting production to cultivars that can survive winter or through production of hybrid varieties. In addition, increased variation in fall and spring weather patterns can lead to frost and freeze damage as vines are preparing for winter in autumn, and also in spring, as buds exit dormancy and tender new growth is present. Frost risk is a constant concern for fruit crop production as young green leaves lack the physical defenses associated with freeze resistance during dormancy (freeze barriers and dehydrated tissues)^1–3^. There is no indication that winter variability will cease, thus there is a great need for better understanding of grapevine chill and freeze exposure response in order to identify candidate genes for future breeding of hybrid varieties suited for these challenging growing conditions.

Studies of freeze shock (<0 °C) and chill shock (4–10 °C) stresses in a number of different crop and plant species have resulted in a common cold response (COR) gene regulation and signaling cascade. While a temperature sensor has yet to be confirmed, plants are able to perceive low temperature and initiate a gene regulation cascade that prepares the plant to tolerate or resist freeze damage. This cascade begins with the activation of calcium signaling, triggering downstream MAPK kinase activity^4, 5^, and changes in the activity of the BHLH transcription factor family *ICE*. These *ICE* genes, in turn, bind to DNA and activate the expression of the cold-binding factor/dehydration responsive element-binding (CBF/DREB) transcription factors^6–8^. *CBF/DREB* genes in turn bind to the DNA and activate many other downstream genes, called *COR* genes, leading to changes in cryoprotectant levels and sugar metabolism, as well as impacts on growth and cell expansion^9, 10^. The elements of this CBF response have been observed across the plant kingdom and several of the key genes have been identified in grapevine^11–13^.

Many plants go through a process called acclimation when they are exposed to low, but non-freezing temperatures. This process is believed to contribute to enhanced freezing resistance by giving the plant time to adjust its physiology to resist the stresses of ice formation, such as changes in membrane structure, alterations in sugar concentrations, and production of cryoproteins^14^. Studies in Arabidopsis have demonstrated an enhancement of freeze tolerance and survival after exposing plants to an acclimating temperature of 4 °C for 24 h. However, a previous study in grapevine demonstrated that pretreatment of swollen buds with the same acclimating temperature was not effective at reducing freeze damage^15^. Few other studies have been conducted to examine the potential resistance of grapevine growing tissues to cold temperatures or to examine variation in freeze response of leaves. The leaves of grapevine are not considered particularly freeze resistant, typically suffering damage at temperatures below −2.5 °C^15^. Studies examining frost protectants have demonstrated that leaves can survive these low damaging temperatures if ice nucleation is prevented through the use of kaolin clay or through eliminating ice-nucleating bacteria^16^. These studies have demonstrated that leaves can survive freezing temperatures “by chance” and sporadic damage in vineyards following a frost are likely due to stochastic avoidance of ice formation and leaf supercooling. It remains unknown whether actual freeze resistance mechanisms and variation between cultivars for leaf freeze resistance occurs. In contrast to the lack of studies in leaf freeze resistance, many studies have been performed examining the cold hardiness of grapevine dormant tissues using methods such as differential thermal analysis to determine lethal temperatures for bud and cane tissues during winter^17, 18^. As a result of these studies, different grapevine cultivars and grapevine species have been ranked as cold hardy or cold tender. The majority of transcriptomic studies examining cold-temperature effects in grapevine involve studies of leaf or berry tissue^19–23^. During these studies, cold stress is often applied as low, non-freezing temperatures such as 4 °C. These studies have demonstrated a interconnected web of gene regulation shared with aspects of drought and salt stresses as well as hormone responses and biotic stress^10^. Cold stress results in the activation of hundreds of genes with the vast majority of their function related to changes in transcription factors and activation of genes with poorly annotated information. These studies are important for understanding the transcriptional response and potential mitigation pathways associated with cold stress damage, but they have not been conducted under freezing conditions. Further complicating the issue, comparisons of cold “hardy” and “sensitive” genotypes may not reflect actual leaf freezing response as these phenotypic designations are due to the resistance of dormant tissues, not green growing tissues.

Studies have been conducted to examine specific gene families (e.g., *WRKY*) involved in cold stress response^24^ or on whole-transcriptome analysis of low-temperature stress in grapevine comparing cultivated and wild species^21^, but no analysis of the transcriptomic landscape has been conducted to elucidate the potential differences between low temperature and freeze stresses in grapevine. Therefore, the objective of this study was to identify transcripts and pathways that are differentially expressed in young leaf tissue, and enriched in response to various cold stresses. We test the hypothesis that exposure to low temperature increases freeze resistance in grapevine using contrasts between temperature exposures to explore the primary transcriptomic processes associated with acclimation/chill (4 °C) exposure, freeze shock (−3 °C), and freeze shock after acclimation (4 to −3 °C). Pathway enrichment analysis was applied to this data to identify the significant changes in gene expression that are shared among and differ between *V*. *vinifera* cultivars.

## Materials and Methods

### Plant Material and Treatments

Five different *V. vinifera* cultivars (“Riesling”, “Cabernet Franc”, “Chardonnay”, “Sangiovese”, and “Tocai Fruliano”) were collected as dormant canes from the field in midwinter (December 2013) and cut into single node cuttings. Cuttings were held in a cold room for 1000 h (~42 days at 4 °C) in order to synchronize dormancy state^25^. Cuttings were then transferred to a growth room with a temperature of 22 °C and a light cycle of 16/8 light/dark to initiate growth and bud burst. When cuttings had reached the E–L stage of 7^26^, cuttings were randomized and placed into styrofoam trays with the basal end encased in parafilm wax and treated with one of four conditions:

1. Control, maintained in growth chamber until collection of tissue for RNA.
2. Acclimated/Chill stress (hereafter: acclimation/acclimated), placed in 4 °C for 48 h, then collected for RNA.
3. Acclimated freeze, placed in 4 °C for 48 h, then exposed to a programed temperature cycle of 4 °C for 30 min, decreasing temperature ramp 0.5°/min to −3.0 °C, hold for 45 min at −3 °C, increasing temperature ramp 0.5 °C/min to 4 °C, hold for 30 min. Tissues were collected for RNAseq at the end of the 45 min hold at −3.0 °C.
4. Non-Acclimated-Freeze (Freeze), no 4 °C pre-treatment, but the same programed freeze exposure outlined above.

Cuttings were lightly misted with dH_2_O prior to being placed in the freezer chamber in order to ensure that uniform freeze occurred across all samples in the chamber and to prevent random leaf supercooling. Six replicate freeze stress runs were performed for the control freeze and acclimation freeze tests and visual damage assessments were performed 4 days after freeze exposure.

### RNAseq Libraries and Quality Control

Leaf tissue was collected in triplicate from E–L 7 stage leaves in each of the treatment conditions and immediately frozen with liquid nitrogen. For tissue collected from freeze treatments, only green tissue without obvious freeze damage (large discolored sectors) was collected to avoid biasing sampling toward cell death. Total RNA was extracted from these tissues using Sigma Spectrum kits (Sigma-Aldrich, St. Louis, MO, USA) and strand-specific libraries prepared^27^. Three replicate samples were extracted and prepared for each cultivar × treatment combination for a total of five cultivars, four treatments, and three replicates. These libraries were multiplexed with barcode adapters and sequenced as 100 bp single-end reads on an Illumina 2500.

Raw reads were demultiplexed based on custom 6 bp barcodes. Residual adapter and low quality sequences were removed using Fastx-tools (fastq_quality_filter –Q33 –q25 –p 25; fastx_trimmer –Q33 –f 7;cutadapt rcprAC rcprBC –minimum-len 25 –O 3). Final read quality was evaluated using FastQC. Quality reads were aligned to the *V. vinifera* 12xV2 genome (https://urgi.versailles.inra.fr/Species/Vitis)) and the V1 annotation (http://genomes.cribi.unipd.it/grape/) using Tophat (tophat -p 48 --library-type = fr-firststrand -o outputfilename -G V1.gff replicate_cultivar_treatment.fq). Alignment statistics can be found in Supplemental Table 1. Uniquely aligned reads were quantified for all V1 annotated genes using HTSeq (htseq-count -f bam -s reverse replicate_cultivar_treatment.bam V1.gff). Normalization and differential expression were completed using the edgeR package following the default workflow with the following exceptions. Library adjusted counts per million (CPM) values were computed and lowly expressed genes were excluded by removing features with CPM <2 and without expression in at least 12 libraries for analyses with cultivars combined, and at least 3 libraries for within cultivar analysis (Supplemental Table 2). Differentially expressed (*DE*) genes were defined based on a FDR corrected *p*-value <0.05 and at least a 2-fold change (Supplemental Data 1). Illumina sequence data are available in the sequence reads archive under the Bioproject number SUB3019356 with accession numbers SAMN07617826-SAMN07617885.

### Pathway and Enrichment Analysis

*DE* gene lists were compiled for treatment contrasts, pooling all cultivars, to produce data sets of up and downregulated genes for each treatment. DE lists for each cultivar were also compiled at the treatment level. After determining *DE* genes, the program VitisPathways^28^ (http://momtong.rit.edu/cgi-bin/VitisPathways/vitispathways2.cgi), was used to identify biological pathways and molecular functions being affected by treatment, leveraging the Vitisnet database (https://www.sdstate.edu/vitisnet-molecular-networks-grapevine)^29^. Enriched pathways of *DE* genes were determined using 100 permutations, a Fisher’s exact test of *p* < 0.05, and a permuted *p*-value of <0.1. Pathways that were enriched both in the combined analysis as well as observed in cultivar × treatment analyses, present in at least four of five cultivars, were determined to represent consistent and necessary stress responsive pathways. These pathways were further investigated to elucidate the specific genes which are differentially regulated under these conditions.

Shared and unique *DE* genes and enriched pathways were visualized with InteractiVenn^30^ (https://omictools.com/interactivenn-tool). Candidate pathways and logFC results for genes within these pathways were visualized using Cytoscape 3.4.0 (http://www.cytoscape.org/).

## Results

### Phenotypic Response of Grapevine Leaves

**Fig. 1:**
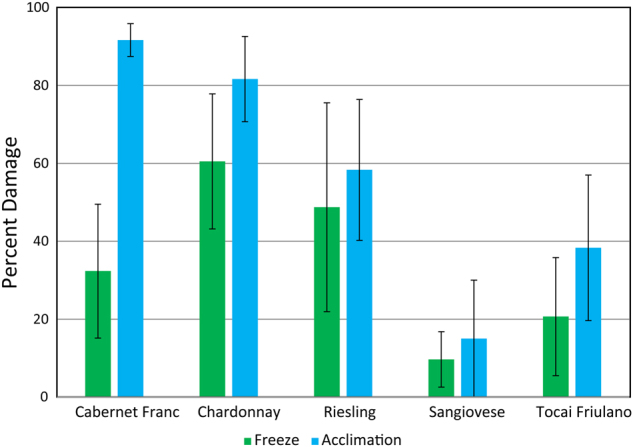
Phenotypic response observed on leaf tissue following freeze treatments. Bars are the average of six replicated experiments, error bars represent standard error of the mean

No apparent damage to leaves were noted in control or acclimated treatment. Following the two freeze treatment conditions, damage was initially noted as leaf tissue with a watery appearance, likely indicating tissue damage and cell leakage. After 4 days of recovery, these damaged regions had dried out and appeared as large lesions. A wide range in damage was observed in freeze-exposed leaves, ranging from the least-damaged cultivar “Sangiovese”, with ~10% damage, to the most-damaged “Chardonnay”, with ~60% damage. All five cultivars in this study had increased damage from freezing following acclimation treatments. “Sangiovese” suffered only slightly more damage relative to control test but “Cabernet Franc” experienced a dramatic increase in freeze damage from ~32% to ~92% after acclimation period (Fig. 1).

### RNAseq Data Quality and Read Count Statistics

The mean size of the sample libraries was 4.6 M reads after QC and alignment. Spearman's correlations were examined for cultivar libraries and a single library was determined to represent an outlier, likely due to insufficient sequenced reads (Supplemental Fig. 1; Supplemental Table 1). This library, replicate 1 for Riesling in warm conditions, was removed from the analysis. A total of 18 367 genes were detected in this study after filtering for low expression values and when analyzed with all cultivars grouped within treatment, representing 61% of the 29 971 genes represented in the Cribi-V.1 gene annotation (http://genomes.cribi.unipd.it/grape/). An MDS plot was used to visually assess the effects of cultivar and treatment on global gene expression patterns. Treatments tended to cluster separately with overlap between acclimation and acclimation freeze treatments while differences driven by cultivar were not apparent in the represented axes (Supplemental Fig. 2).

### Differential Expression

**Fig. 2:**
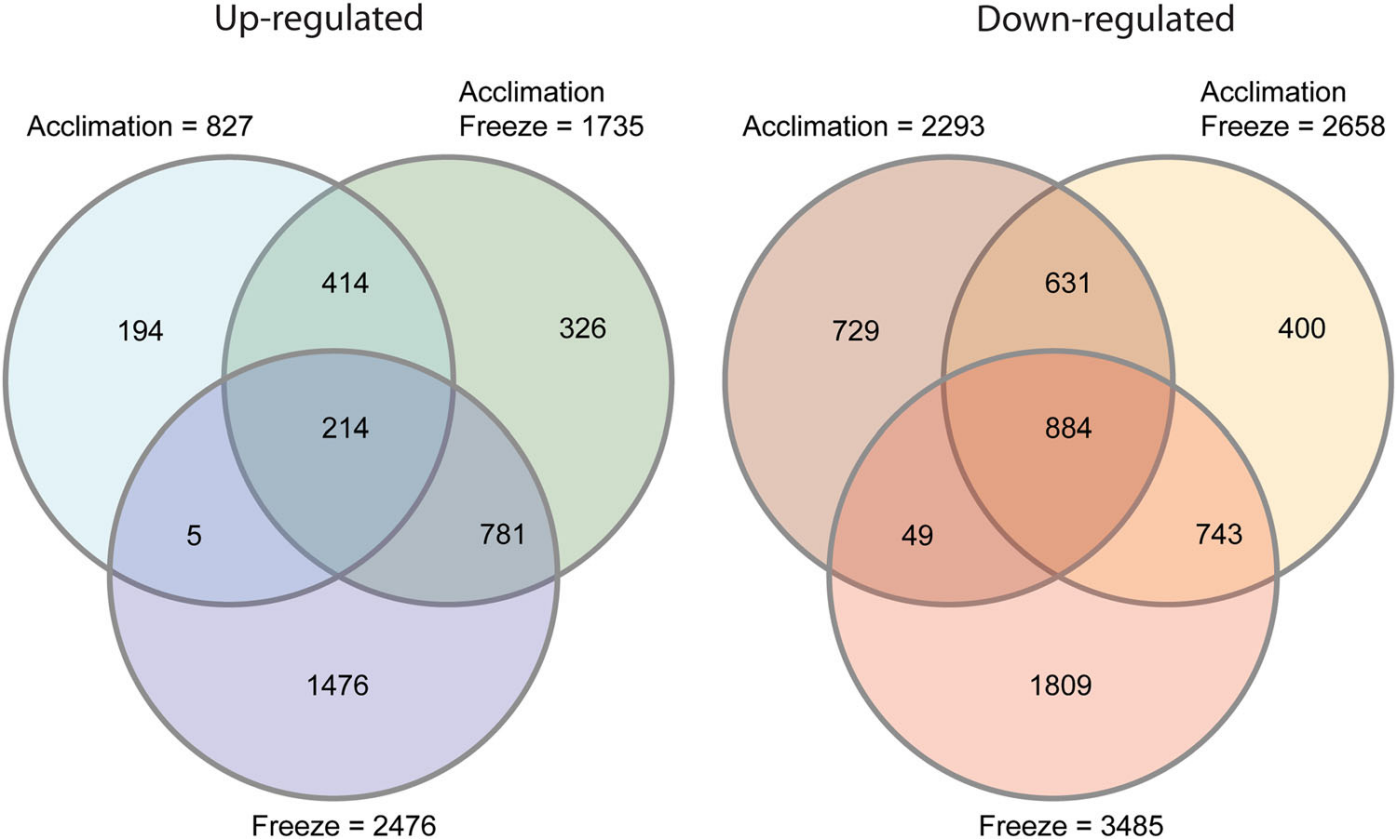
Differentially expressed genes based on treatment. Venn diagram of shared upregulated and downregulated *DE* genes in acclimation and freeze stress treatments

Contrasts between warm and stress treatments revealed 8070 genes that were differentially expressed at LogFC >1 and FDR <0.05. In general, stress treatments resulted in a greater number of genes that were downregulated rather than were upregulated. Hierarchical clustering of samples using differentially regulated genes are able to delineate treatment affects. Acclimation freeze samples are found to cluster both with freeze and with acclimation treatments, demonstrating overlap between responsive genes in these treatments (Supplemental Fig. 3). Acclimation stress resulted in the least number of *DE* genes and freeze treatments resulted in the greatest number. Stress treatments resulted in 214 shared upregulated genes and 884 shared downregulated genes. For upregulated genes, acclimation and acclimation freeze shared 414 upregulated genes and 631 downregulated genes while acclimation freeze and freeze shared 781 genes and 743 genes respectively. Only 5 genes were observed to be upregulated in both acclimation and freeze treatments and 49 were shared as downregulated (Fig. 2). Lists of shared *DE* genes grouped by treatment with functional annotation can be found in Supplemental Data 2. Counts of *DE* genes for treatment averages and for cultivar specific contrasts demonstrate a similar pattern of high *DE* genes for freeze treatment comparisons and low *DE* genes for acclimation treatment (Supplemental Table 3).

### Pathway Enrichment

Results of the pathway analysis demonstrated that there were an abundance of enriched pathways in the process categories of metabolism, environmental processing, and transcription factors, and a reduced enrichment in processes of transcription, cellular processes and transport (Table 1).

**Table 1:** Sample of the pathways and processes with significant enrichment of *DE* genes, partitioned for up and downregulated genes in stress

Pathway or process	Acclimation		Freeze		Acclimation freeze	
	Down	Up	Down	Up	Down	Up
1. Metabolism	11	11	9	29	17	13
1.1 Carbohydrate metabolism	2	2	1	6	2	2
1.3 Lipid metabolism	0	4	2	4	3	2
1.5 Amino acid metabolism	1	3	1	7	1	4
1.9 Biosynthesis of secondary metabolites	5	1	0	7	2	4
2. Transcription	2	2	2	0	2	0
3. Environmental processing	4	3	2	4	3	5
3.2 Hormone signaling	3	2	2	2	3	3
4. Cellular processes	3	1	2	0	3	0
5. Transport	2	4	3	5	4	3
6. Transcription factors	12	8	15	15	11	18

Pathways and processes follow VitisNet designations. Numbers in columns indicate the number of pathways within the classification that were identified as enriched.

Further investigation within these stress-responsive categories revealed overrepresentation of subcategories of carbohydrate, lipid, and amino acid metabolism, as well as biosynthesis of secondary metabolites and pathways associated with hormone signaling. A large number of *DE* genes were enriched in different transcription factor families, within both up and downregulated responses. Comparing the effects of stress, averaged across cultivars, and by also checking for enrichment within cultivar, allowed us to identify candidate pathways that were consistently reactive to stress treatments. Of the many enriched pathways detected within this experiment, we identified several key pathways that were repeatedly upregulated and represented among comparisons of stress treatments and shared among cultivars. (Supplemental Data 3 and 4). These enriched pathways are described below. LogFC information and *Vitis* gene loci represented in these pathways can be found in Supplemental Data 5-10.

#### Ethylene

**Fig. 3:**
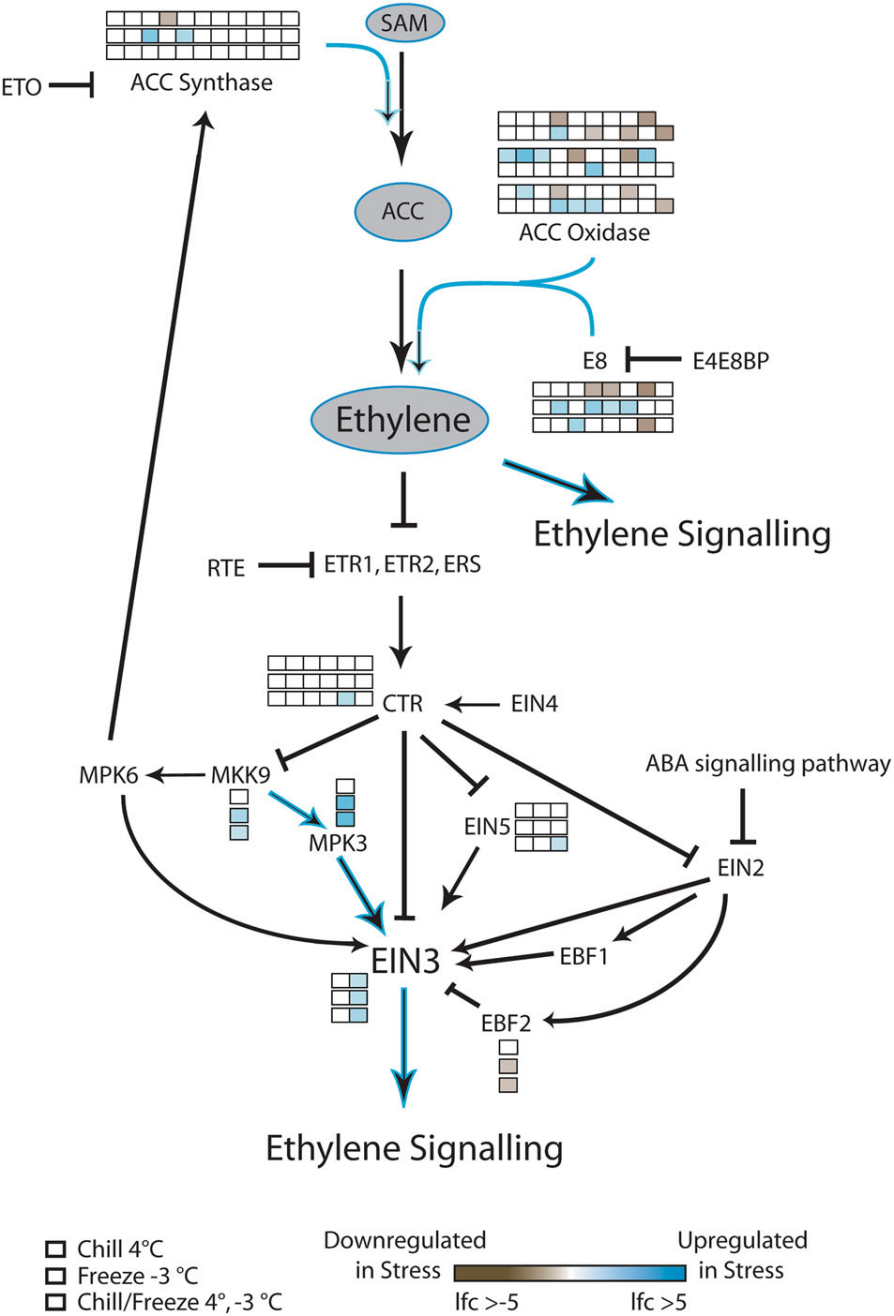
Reduced Vitisnet ethylene synthesis and signaling pathway demonstrating log fold change in differentially expressed genes as a result of stress treatment. Each box in a series represents a cribi-V1 annotated paralog for the indicated gene. Genes without boxes were not differentially expressed in this experiment

The transcriptional landscape of the ethylene pathway, including ethylene synthesis, signaling, and downstream transcription factor activation was very different between acclimation stress and freeze stresses (Fig. 3). Genes in the ethylene production pathway were upregulated in freeze and acclimation freeze treatments, suggesting that ethylene levels may increase in the leaves. Negative regulators of ethylene signaling downstream of ethylene synthesis were not upregulated.

#### Abscisic Acid (ABA)

**Fig. 4:**
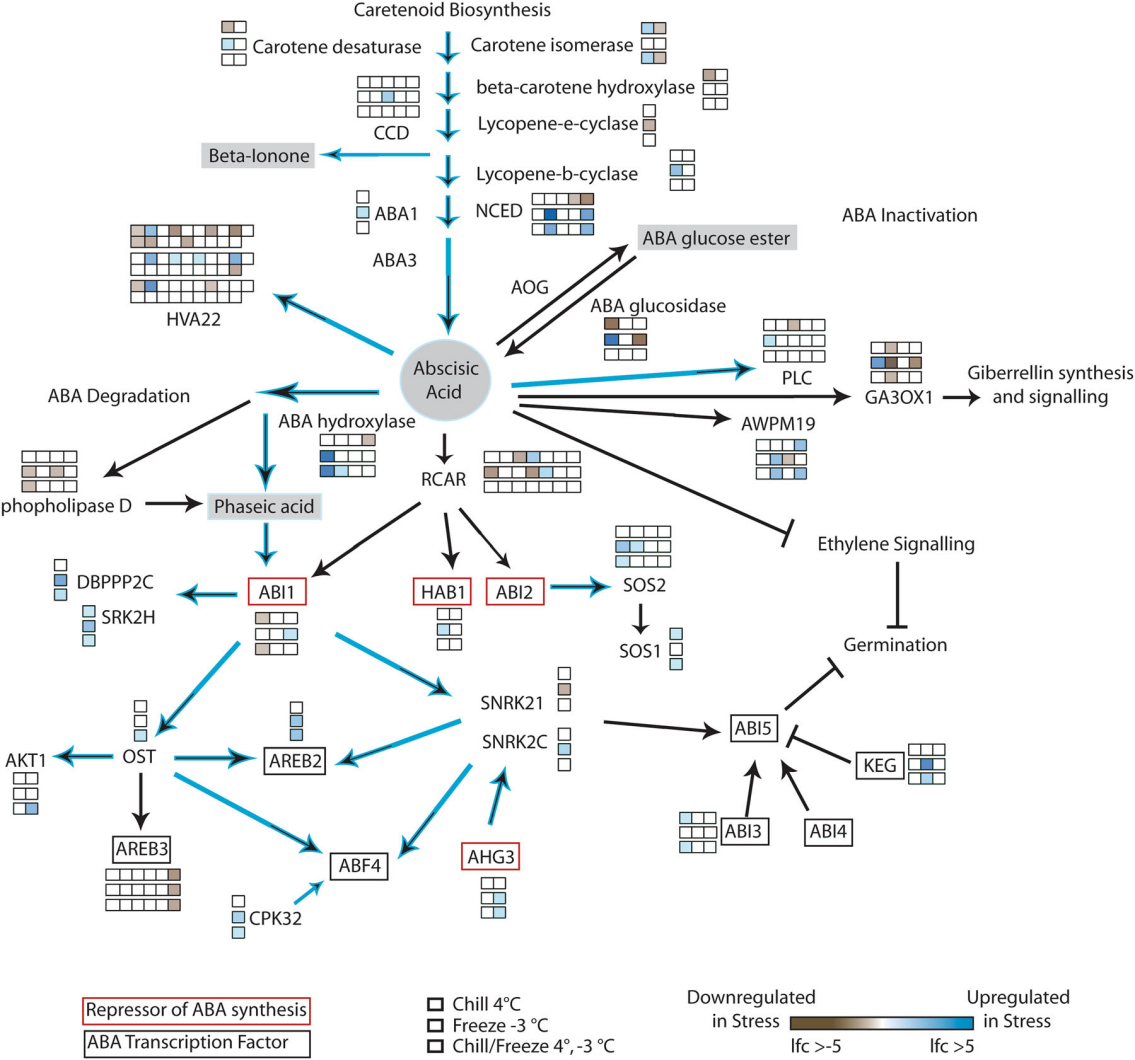
Reduced Vitisnet ABA synthesis and signaling pathway demonstrating log fold change in differentially expressed genes as a result of stress treatment. Each box in a series represents a cribi-V1 annotated paralog for the indicated gene. Genes without boxes were not differentially expressed in this experiment. Blue arrows indicate patterns of differential expression associated with freeze stress. Genes that repress ABA synthesis shown in red boxes. ABA-dependent transcription factors in black boxes

Enrichment of three pathways was consistently observed across temperature treatments that represent components of abscisic acid (ABA): carotenoid biosynthesis (secondary products), ABA biosynthesis, and ABA signaling (Fig. 4). The effect of freeze stress is strongly seen in the expression of NCED3, the committing step in ABA synthesis^31^. Clear differences were observed between acclimation and freeze stresses, particularly in the downstream signaling aspects leading to ABA-based transcription factor activation. Since ethylene signaling was not seen to be repressed (Fig. 3), the interaction between ABA and ethylene seems to be a coordinated, rather than antagonistic interaction leading to large changes in downstream stress responsive transcription factors.

#### NAC, WRKY, AP2/ERF

**Fig. 5:**
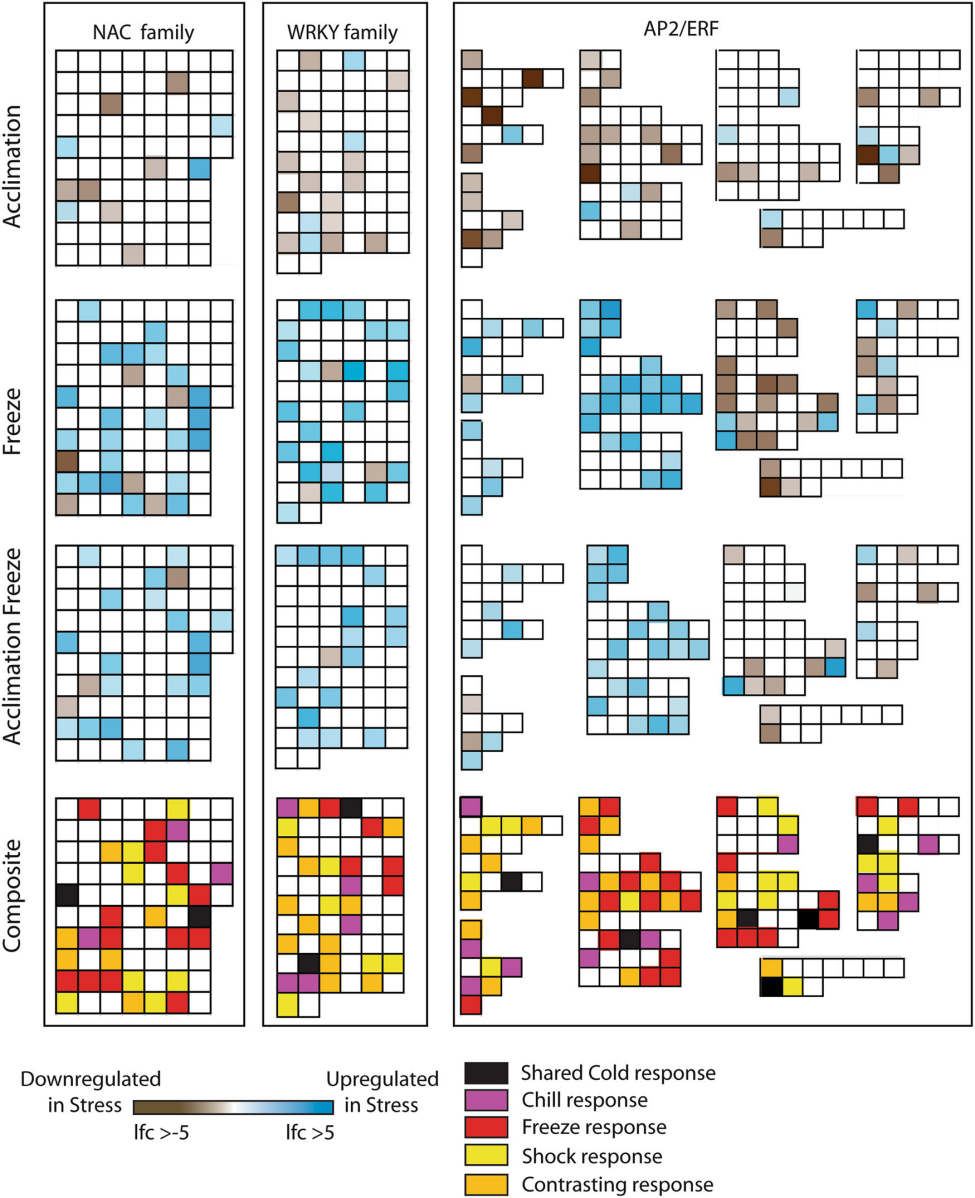
NAC, WRKY, and AP2/ERF transcription factor families demonstrating log fold change in differentially expressed genes as a result of stress treatment. Each box in a series represents a cribi-V1 annotated gene. Composite image compiled to demonstrate shared, contrasting, and unique responses of *TF* genes

Several transcription factor families were significantly enriched in this experiment including the HSF, PLATZ, MYB, GRAS, NAC, WRKY, and AP2/ERF families. Of these, the NAC, WRKY, and AP2/ERF families were specifically enriched for genes that were upregulated in stress treatments (Fig. 5). For the NAC and WRKY families, the signature of stress response was very similar. In acclimation stress, there were a few *DE*, upregulated genes, but a greater number of downregulated genes. In contrast, freeze stress resulted in a much greater number of upregulated genes in these gene families and acclimation freeze responses were typically lower in fold change than that of freeze stress.

Major changes in the *AP2/ERF* gene family were observed between acclimation and freeze treatments with acclimation freeze resulting in a similar, but muted pattern relative to freeze shock. The composite portion of Fig. 5 demonstrates the genes which were related to specific contrasts in this experiment; “Cold” (same logFC pattern in all cold treatments), “Chill” (associated with acclimation treatment), “Freeze” (shared regulation in both freeze treatments), “Shock” (only associated with freeze shock), and “Contrast” (regulation was in opposite directions in stress). The relative proportion within these categories indicate that shared responses only represent around 6–8% of the observed gene expression changes with “Freeze”, “Shock”, and “Contrast” each representing the dominant patterns of expression.

#### Sugar Metabolism

**Fig. 6:**
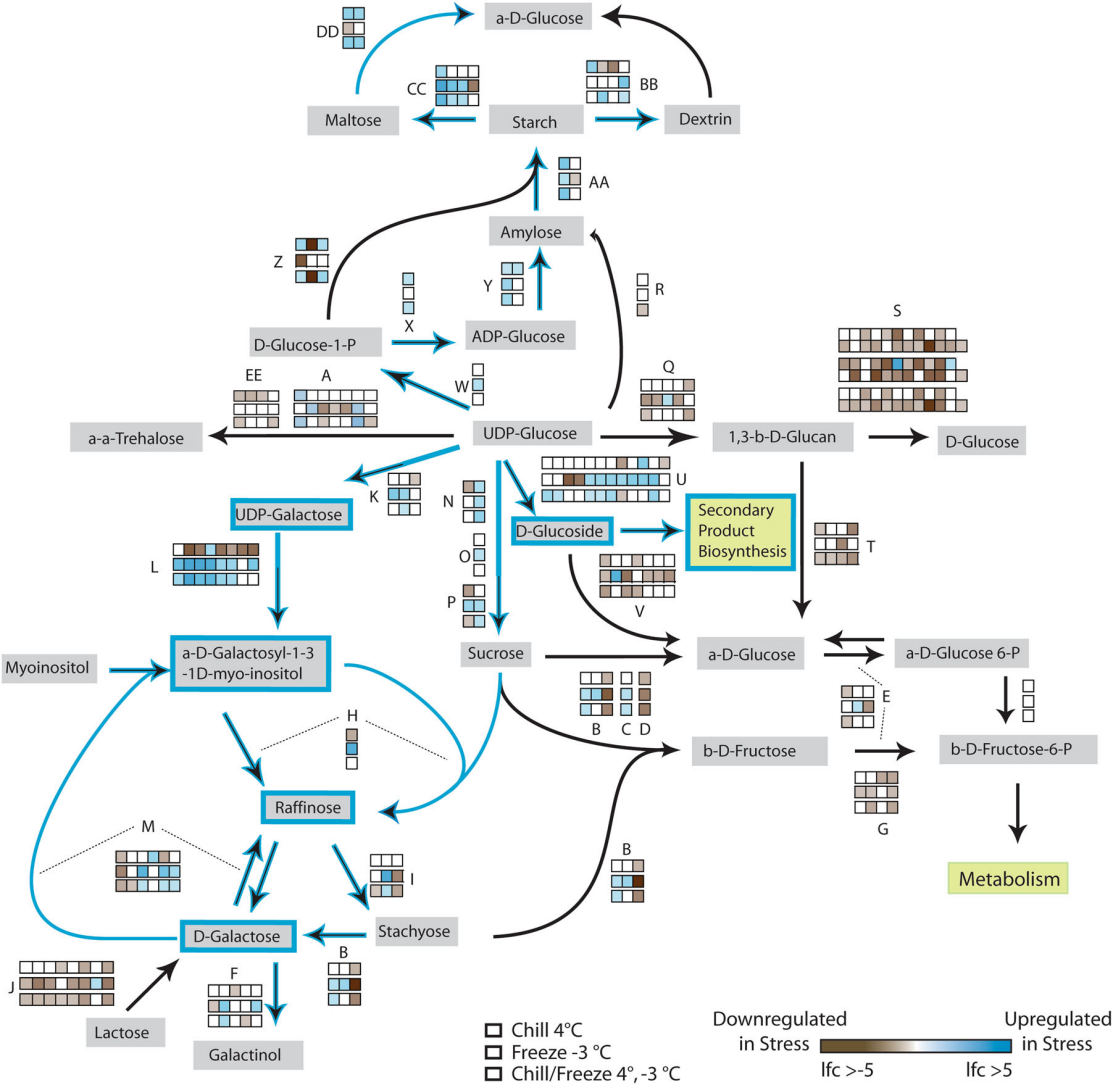
Sucrose, starch, and galactose pathways demonstrating major shifts in the expression of metabolic enzymes in response to chill and freeze stresses. Metabolic enzymes denoted with letters for brevity (supplemental data 10). Blue indicates significant fold upregulation, brown indicates significant downregulation. Blue directional demonstrate presumed change in metabolites under freeze stress. Gene family members with no log fold change not presented for brevity

The pathways associated with sugar metabolism, specifically the starch, sucrose, and galactose pathways, were significantly enriched for *DE* genes. When examining the interaction of these complex pathways (Fig. 6) we observed that all stress treatments resulted in major downregulation of genes leading to the production of d-Glucose (*Q* and *S*), d-Glucose 6-Phospahte (*D*, *E*, and* V*), d-Fructose 6-Phosphate (*D*, *E*, and *G*), as well as the degradation of Lactose (*J)* and Trehalose (*A* and *EE*). All stress treatments also seemed to trigger enzymes responsible for production of Amylose, Starch, Maltose, and Dextrin (*W*, *X*, *Y*, *R*, *AA*, *BB*, *CC*, and *DD*). Specific differences were observed in the response of acclimation versus the two freeze stress treatments. These treatments resulted in differing expression patterns for genes involved with the conversion of UDP-galactose to α-d-galactosyl-1,3-1D-myo-inositol, a precursor to raffinose, and in the conversion of UDP-glucose to d-glucoside, a common precursor that enters into many secondary product biosynthesis pathways. The transcriptional landscape of these sugar pathways thus demonstrate a shunt away from growth-based metabolic substrates like glucose and fructose in control leaves, toward that of storage (starch) in chill stressed leaves, and toward storage, osmotic protection (raffinose), and defense response (secondary products) in freeze stress leaves.

### Phenotype and Pathway Integration

When examining the phenotype of freeze resistance, many differences between the five cultivars studied could be observed. A list of the top upregulated and downregulated genes as well as those genes found consistently is reported in Supplemental Data 3. Of particular interest in this study was “Sangiovese”, which appeared to resist freeze damage both with and without acclimation treatment. We examined all upregulated genes across the five cultivars in both freeze and acclimation freeze treatments by cultivar and identified the genes which were uniquely differentially expressed in “Sangiovese” in order to determine if any pathways were overrepresented and might be linked to increased freeze resistance. 567 DE genes were unique to “Sangiovese” and these genes were shown to result in 16 significantly enriched pathways in *Vitis* (Supplemental data 11). Of particular interest is the phenylpropanoid biosynthesis pathway which had “Sangiovese” specific transcripts for phenylalanine ammonia lyase and stilbene synthase, the committed steps leading to the production of stilbenes, secondary products associated with stress response. Of the 42 predicted stilbene synthase genes in the *Vitis* annotation, 24 were upregulated within freeze and 15 were upregulated within acclimation freeze treatments in “Sangiovese”.

## Discussion

Several different studies have been conducted looking at the effects of low temperature on the grapevine leaf transcriptome. Transcriptome differences have been evaluated in comparisons between low temperature and other abiotic stresses like drought or salt^19^, between species with varied cold hardiness phenotypes^22, 24^, or looking at specific gene families^21^. In each of these studies, the applied cold stress is that of a low temperature, about 4–5 °C. This is the same temperature used as an acclimation temperature in other crop and model systems to prime a plant to develop increased freeze hardiness. Since most designations of hardiness in grapevine describes the freeze resistance of dormant tissues such as buds and canes, comparisons of hardy versus sensitive grape varieties at low, non-freezing temperatures in leaf tissue may not completely elucidate the important genes and pathways for freeze resistance in grape. In this study we wanted to better understand how the transcriptomes of a leaf exposed to low temperature compared with that of a leaf exposed to simulated frost/freeze conditions to determine whether acclimation of growing tissues leads to enhanced freeze resistance. In addition, we wanted to lay the foundation of the grapevine freeze stress transcriptome for future studies of dormant tissue response.

### Phenotypic Response to Stress Treatments

In this study, cuttings were exposed to warm control conditions at 22 °C until they reached the E–L stage 7, a stage where field damage due to frost is commonly observed. No leaf damage was observed in these control conditions, nor on cuttings exposed to low temperature (4 °C). This was in stark contrast with cuttings treated with a simulated frost/freeze event which showed varied evidence of freeze damage as large sectors of leaves appearing to be dark in color and water saturated. Presumably, these sections were parts of the leaf where cell membranes had ruptured and cellular fluids had saturated the tissues. When cuttings were first acclimated at 4 °C for 48 h and then treated with the simulated frost/freeze, greater levels of damage were observed, refuting the hypothesis that acclimation treatment would decrease freeze damage in grapevine. This result is in contrast to studies in Arabidopsis^32^, where low-temperature treatment primes freeze resistance and leads to greater freeze survival. However, it should be noted that a similar result was observed for two grapevine cultivars “Siegrebbe” and “Madeleine Angevine” tested at a much earlier phenological stage than the cuttings in our study^15^. This finding suggests that the transcriptional, protein, or metabolic response to low temperature is not an adaptive change toward greater freeze resistance in grapevine and that the current methodology for determining candidate genes and pathways from cold “hardy” and “sensitive” varieties using low non-freezing temperatures should be reevaluated. In fact, this commonly used cold stress treatment seems to uncover the transcriptional response that contributes to reduced freeze resistance. It is possible that this result partially explains why some studies show that cold sensitive grapevine cultivars have greater changes in gene expression when compared with tolerant varieties under low-temperature stress^21^.

### Transcriptional Reprogramming in Response to Stress

More than 18 000, genes of the V1 annotation were detected in this study as being expressed in grapevine leaf tissue. Acclimation treatments resulted in the smallest number (3 120) of *DE* genes compared to the control. Acclimation freeze and freeze treatments resulted in 1.5 × (4 393) and 2 × (5 961) as many *DE* genes as acclimation, demonstrating a more dynamic alteration in the transcriptome when leaves are exposed to freezing stresses, even when freeze exposure is short (<1 h).

We chose to target our analysis of the data set to a defined subset of biologically relevant pathways and gene families that were consistently observed across treatments and cultivars. To our knowledge, this is the first evaluation of the grape leaf transcriptome under these stress conditions and the patterns observed in this data now provide a foundational basis for testing hypotheses of putative metabolites for freeze response in grapevine leaves.

### Ethylene

The results of this study repeatedly demonstrated that pathways related to the synthesis and signaling of ethylene and ABA were enriched for *DE* genes, consistent with other studies in grapevine examining the roles of these hormones in abiotic stress response^33, 34^. The dominant contrast observed for the ethylene pathway occurred between acclimation and freeze treatments. Acclimation treatments resulted in general downregulation of ACC synthase and oxidase genes, as well as homologs for E8, the results of which could be reduced production of ethylene. *Vitis* *EIN3* genes were upregulated in acclimation, suggesting that signaling from this portion of the ethylene pathway is utilized in acclimation conditions. In contrast, all aspects of ethylene production through ACCase and ACC oxidase, as well as MKK9, MPK3, and EIN3 mechanisms were upregulated in freeze conditions and EIN3 repression by EBF2 was downregulated. These taken together suggest that while ethylene signaling may be low in acclimation treatment, there appears to be a rapid and upregulated role in response to freeze stress. It should be noted that studies that have examined the ethylene pathway in grape exposed to acclimation stress observed increases in ACCase and ACC oxidase, as well as boosted ethylene at earlier time points (8 h post exposure), but returning to control levels by 48 h^23^, which is in agreement with our results. Taken together, our results demonstrate that with or without acclimation pre-exposure, the ethylene pathway is rapidly stimulated in leaf tissue following exposure to freezing stress conditions.

### Abscisic Acid

ABA synthesis and signaling were impacted in a similar way to ethylene. Up and downregulation of genes like carotene isomerase were observed in acclimation and acclimation freeze treatments while downstream genes were downregulated in freeze, suggesting that there is not a concerted recruitment of the early genes leading to ABA synthesis. NCED3 was downregulated in acclimation, but was greatly upregulated in both freeze and acclimation freeze, and ABA1 was upregulated in freeze shock, driving ABA synthesis from existing metabolite pools. Continuing downstream, freeze treatments increased the expression of genes responsible for ABA degradation into phaseic acid and subsequently toward the activation of the protein phosphatase 2C gene, *ABI1*. ABI1 was upregulated in freeze, but downregulated in acclimation and acclimation freeze, suggesting that acclimation treatment in some way buffered *ABI1* gene expression. Previous studies have demonstrated that loss of function of ABA1 and/or ABI1 results in lower ABA synthesis and perception, reduced expression of cold-regulated genes, and also correlates with loss of freeze resistance in *Arabidopsis*^10^, which could explain the increased damage we observed in acclimation freeze treatments. Downstream of ABI1, upregulation of *OST* and *SNRK2* genes as well as upregulation of CPK32 leads to the activation of *AREB2* and *ABF4* (*AREB4*) genes indicating that ABA-responsive transcription factors are increased under freeze treatments. Several repressors of ABA synthesis were differentially modulated by acclimation vs. freeze, with ABI1 being an example but also AHG3, which was up in freeze but not significantly changed in acclimation. Acclimation-related and freeze-related gene expression of SOS1, SOS2, ABI3, and KEG also demonstrate that acclimation and freezing stresses result in very different overall patterns of gene expression in ABA synthesis and downstream ABA-dependent gene regulation.

### Transcription Factor Families

Downstream of the ethylene and ABA pathways lay many different stress-associated transcription factors, which are in part activated by these hormones including the NAC, WRKY, and AP2/ERF transcription factor families^35^. The NAC transcription factor gene family has been typically associated with the regulation of plant growth and development, as well as biotic and abiotic stress response^36^. In this study, we detected significant differential expression of 32 of the potential 75 annotated NAC family members in the Vitisnet/V1 annotation. The expression patterns of the *NAC* genes were quite different between acclimation and freeze treatments. In acclimation, 11 NAC members were differentially expressed, of which 7 were downregulated and 4 were upregulated. A previous study described 8 *NAC* gene family members with differential expression in previous studies of cold stress, and it is interesting that our results mirror those of these earlier stress studies^36^. In contrast, a major change in the transcriptional landscape occurred following freeze treatments; 28 NAC members were differentially expressed, and the vast majority represented upregulated genes. Fifteen of these NAC had similar fold change in freeze and acclimation freeze treatments, indicating many *NAC* genes respond to freeze stress with or without low temperature pre-treatment. Of the NAC family members, only *VvNAC18* and *VvNAC26* were seen to be regulated in the same manner in all three cold treatments. *VvNAC26* was previously observed to have the greatest expression change in response to abiotic stress treatments in a family-wide analysis, and our results support this as a general *COR NAC* gene. *VvNAC18* was seen to be inflorescence specific, which does not agree with the analysis here but it is also important to note the large increase of *DE* *NAC* genes in response to freeze, demonstrating a much wider role for *NAC* genes in cold stress response than previously revealed^36^.

The WRKY transcription factor gene family was also enriched in our study, and this family has been associated with the regulation of the transcriptome in response to biotic and abiotic stresses and a number of studies have examined this family in grapevine^24, 37^. Many of the *WRKY* genes also seem to play a role regulating the cross-talk associated with plant hormone responses to stress, with many of the gene family members responding to ABA, ethylene, jasmonic acid, and salicylic acid exposure^38^. Previous studies of *WRKY* gene expression detected by microchip experiments or reverse transcription–PCR in response to cold temperatures have been conducted using the acclimation temperature used here and identified a number of cold responsive *WRKY* genes^24, 37^. The gene nomenclature^39^ used in these two studies do not correspond, but correlating the results of these studies suggest many of the same genes respond to cold stress. In our study, the expression pattern differences between acclimation treatment and the two freeze treatments largely mirrored that of the previous studies. Thirty *WRKY* genes were differentially expressed in this experiment in at least one of the cold temperatures. In acclimation treatment, 18 genes were *DE*, 4 upregulated and 14 downregulated. In freeze and acclimation freeze treatments, the number of genes increased dramatically and the vast majority were seen to be upregulated. Only two of the *DE* genes were shared among all three cold treatments, corresponding with *VvWRKY28* and *VvWRKY21* from previous studies^24^. Both of these genes were seen to respond to ABA treatment, suggesting interaction between the ABA results here and *WRKY* gene expression. It is clear from our study that the transcriptional landscape and downstream signaling dependent on WRKY expression is very different between acclimation and freeze treatments.

The AP2/ERF transcription factors make up one of the largest transcription factor families in plants and are characterized by having at least one AP2 DNA-binding domain. This gene family is both large and highly diverse in gene action, with some genes typically associated with growth and development of cells and plant organs, such as the *AINTEGUMENTA*^40, 41^, *SHINE*^42^, and *WRINKLED* genes^43^, and other genes which play roles in the perception of plant hormones and response to biotic and abiotic stresses like *CBF/DREB* and *RAP2* genes^44–47^. The transcriptional pattern of *AP2/ERF* gene expression was highly divergent between acclimation and freeze stress treatments. In acclimation treatment, the vast majority of genes in this family were downregulated in both the growth-related and stress-related modules including *Vitis* CBF genes, which have been shown to respond to cold stress and regulate downstream cold responses. Three of the *Vitis* CBF genes have been shown to be responsive to cold, drought, and other stress treatments in previous studies^11^. However, *VvCBF4* has been found to respond specifically to acclimation stress (4 °C), with its expression increasing quickly after exposure to acclimation temperatures before stabilizing through 8 h of exposure^12^. *VvCBF1* was not detected in our study and *VvCBF2* and *VvCBF3* transcripts were detected but at levels below our expression cutoff, suggesting that none of these multi-stress responsive genes play a major role in grapevine leaf cold stress response. However, it should be noted that *Vitis* CBF genes may be under different temporal regulation and could explain the lack of expression noted here^3^. This result also does not agree with previous studies^11^ but could represent variation between the cultivars or clones used in this study, as has also been shown for cold response of *VvCBF2* in the cultivar “Koshu”^48^. *VvCBF4* was greatly downregulated in the acclimation treatment relative to control expression levels (logFC = −5.69). Although this is in disagreement with the peak in expression seen previously^12^, our samples were collected after 48 h of exposure. Therefore, it appears that early signaling through the *VvCBF4* regulon diminishes over time.

The activity of the *AP2/ERF* genes drastically changes in freeze and acclimation freeze treatments. It is under these conditions that we see a clear difference in the gene regulation between *AP2/ERF* genes governing stress response versus growth and development. The freeze treatment resulted in upregulation and large fold changes in the abiotic stress-associated modules and downregulation and large fold reductions for growth-related modules. This suggests that the perception of imminent freezing results in reprogramming of the *AP2/ERF* gene family and, consequently, downstream genes. The landscape was also similar in both freezing conditions, though acclimation freeze treatments represent a subset of the total freeze response genes and the logFC of these genes are generally lower than those in freeze. These results suggest that the rapid and dynamic changes that occur when freeze shock conditions occur, are in some ways prevented or not needed if grapevine is exposed to lower temperatures prior to freezing conditions. Upregulation of *VvCBF4* was observed in both freeze and acclimation freeze conditions, though the changes were only significant between control treatment and acclimation freeze treatment (logFC = 1.61). This result is interesting as it demonstrates a potentially nuanced regulation of *VvCBF4* by varied cold exposure. As mentioned above^12^, *VvCBF4* typically increases initially when exposed to acclimation temperatures, but then decreases to control levels or lower as seen in this study, by 48 h. We show that *VvCBF4* expression increases as a result of freeze shock, but being pre-treated with acclimation temperature results in a much higher, significant upregulation of this master controller. The reason for this primed effect are not clear, but we can speculate that other aspects of gene signaling that occur upstream of *VvCBF4* may be also primed and could enable greater activation of this cold regulon should freeze conditions occur. It is clear from these results that stress response and gene regulation of the *AP2/ERF* family is drastically different between acclimation and freeze treatments. Many of the genes identified as upregulated in both freeze and acclimation freeze represent new candidate genes that can be investigated for potential use to increase the freeze resistance in grapevine.

### Sugar and Starch

Downstream of plant hormone signaling and the transcription factors that respond to stress are a multitude of metabolism and biosynthesis pathways that play a role in modifying plant physiology to resist or tolerate cold stress. Sugars have long been associated with increases in chill and freeze resistance in a number of crops species^49, 50^. Soluble sugars like sucrose, glucose, fructose, and raffinose have been shown to increase in concentration when plant tissues are cold stressed. These sugars are thought to provide freeze protection through stabilization of membranes, scavenging of reactive oxygen species, acting as signaling molecules, and by decreasing the freezing point as compatible osmolytes^51, 52^.

Studies conducted to measure the carbohydrate fluxes of grapevine leaves exposed to acclimation stress have shown that these sugars do increase over time with glucose, fructose, and sucrose increasing initially and raffinose and galactinol increasing after several days of exposure^53^. We observed that *DE* genes were enriched for the sucrose and starch metabolism and galactose pathways. Unlike the contrasting effects of acclimating temperatures versus freeze temperatures observed for ABA, ethylene, and transcription factors, the pattern of gene expression for several aspects of these pathways were shared. All cold treatments resulted in the upregulation of enzymes of starch synthesis compared to the control, suggesting a general cold stress strategy of increasing non-soluble sugar reserves and this is consistent with other cold stress studies where starch levels were measured after cold (4 °C) exposure^53^. Interestingly, genes that encode enzymes responsible for the degradation of starch into maltose and dextrin were also upregulated, suggesting starch may not be the terminal response to cold. Genes responsible for the production of sucrose were also upregulated in all cold treatments demonstrating that sugar reserves may be partitioned between non-soluble and soluble sugar forms. The relative levels of gene expression change tended to demonstrate that freeze shock stress resulted in greater downregulation than that of acclimation and acclimation freeze. Taken together, all cold stresses had the effect of downregulating the production of simple soluble sugars that are used in general metabolism, likely resulting in the reduction in growth and development that often occurs at cold temperatures.

In contrast to these shared DE patterns, a few key changes in gene expression suggests that these sugar pathways are utilized differently between acclimation and freeze stresses. In both freeze treatments, the expression pattern of phenol-β-glucosyltransferase was upregulated. This enzyme catalyzes the hydrolysis of UDP-glucose to d-glucoside, a general glucose based sugar backbone used in a multitude of pathways related to secondary product biosynthesis, such as flavanols, terpenes and stilbenes. Upregulation of the portion of the pathway leading to the production of raffinose was also seen in response to freeze treatment, in particular the committing step of raffinose synthesis. While we did not directly measure raffinose concentrations in this study, it is clear from our data that freeze induces all the genes leading from simple soluble sugars to raffinose. Increased raffinose concentrations and production has been seen to be a common cold stress response in a number of different plant systems including both perennials and annual species^54, 55^. However, it has also been seen that raffinose itself is not necessary for freezing tolerance as mutant Arabidopsis lines with lack of raffinose synthase are still able to survive freezing conditions^56^. Raffinose could have many different cryo-protective roles, ranging from ROS scavenging to stabilization of photosynthetic proteins^57^. In grapevine, increased levels of soluble sugars, including raffinose, are correlated with changes in dormant tissue cold hardiness^58^ but no direct mechanism has been uncovered. Treatment of grapevine leaves to moderate chill stress of 15/7 °C day/night temperatures increased leaf raffinose concentrations, and that more “hardy” varieties accumulated a higher concentration^59^. All data to date are correlative suggesting that raffinose concentration is tied to cold stress resistance, and this study further supports a role for raffinose in cold resistance. It is clear from our data that there are fundamental differences in the gene regulation and signaling of the carbohydrate pools between acclimated and freeze shock exposed grapevine leaves.

### Cultivar responses

Our experiment clearly demonstrated that low temperature pretreatment of grapevine cuttings does not increase resistance to freezing stress. The phenotypic range of freeze damage was variable between cultivars and similarly, preventing a simplistic assessment of what unique aspects of gene regulation contributes to increased freeze resistance. The number of significant DEs in each cultivar did not correlate with observed leaf damage. Both “Cabernet Franc” and “Sangiovese” had a high number of DE genes in all treatment contrasts yet represent very different phenotypes. As “Sangiovese” seemed resistant to freeze damage both in freeze and acclimation freeze treatments, we explored which genes and pathways were upregulated, enriched, and unique for “Sangiovese”. Of particular note was the phenlypropanoid pathway, and important pathway in secondary metabolism. Stilbenes are a class of plant protective gene products called phytoalexins and have been shown to be important for plants exposed to a multitude of abiotic and biotic stresses^60^. While a few stilbene synthesis genes were upregulated in “Cabernet Franc” (9), “Chardonnay” (8), and “Riesling” (7) in freeze or acclimation freeze stresses, 24 of 42 stilbene synthase genes were upregulated in the “Sangiovese” samples and 7 were uniquely found in “Sangiovese”. Stilbene induction under cold temperature exposure has not previously been investigated and the transcription factors responsible for stilbene synthase expression are unresolved. However, stilbenes were seen to be induced by other stresses that induce ABA and ethylene pathways^60^ and stimulation here may be a result of the changes seen in these hormone pathways. Unfortunately, it is not possible to determine if stilbenes are the reason for increased freeze resistance in “Sangiovese” or if it simply has greater basal expression of these genes, but this suggests that the role of stilbenes in freeze response may be worth further study.

## Conclusion

Young grapevine tissues are faced with the possibility of freezing stress as a result of frost events and resistance to this stress is important to prevent damage in the vineyard. Our study showed that cultivated grapevine appears to have no acclimation ability in green tissues such that pre-treatment of leaves to low, non-freezing temperatures (4 °C) increased damage when leaves were exposed to a short duration freeze treatment. Our study indicates that examination of grapevine cold stress response at 4 °C many not fully elucidate or identify the most important aspects of freeze response. Our results demonstrate that the transcriptional landscape of cold stress at 4 °C and that of freezing stress at −3 °C are very different in key hormone, transcription factor, and sugar pathways As with any assessment of transcriptomic response, future studies are needed to validate the changes in gene expression seen here with metabolomic profiling as well as further investigation into the potential role of stilbenes in freeze response.

## Electronic supplementary material

Supplemental Figure 1

Supplemental Figure 2

Supplemental Figure 3

Supplemental Data 1

Supplemental Data 2

Supplemental Data 3

Supplemental Data 4

Supplemental Data 5

Supplemental Data 6

Supplemental Data 7

Supplemental Data 8

Supplemental Data 9

Supplemental Data 10

Supplemental Data 11

Supplemental Table 1

Supplemental Table 2

Supplemental Table 3
