# Supplementary material for: Divergence in the transcriptional landscape between low temperature and freeze shock in cultivated grapevine (Vitis vinifera)
Source: Hortic Res. 2018 Mar 1;5:10. doi: 10.1038/s41438-018-0020-7 (PMC5830407; doi:10.1038/s41438-018-0020-7)
Supplement: Supplementary file 1 — Supplemental Figure 1 [file 41438_2018_20_MOESM1_ESM.pdf]

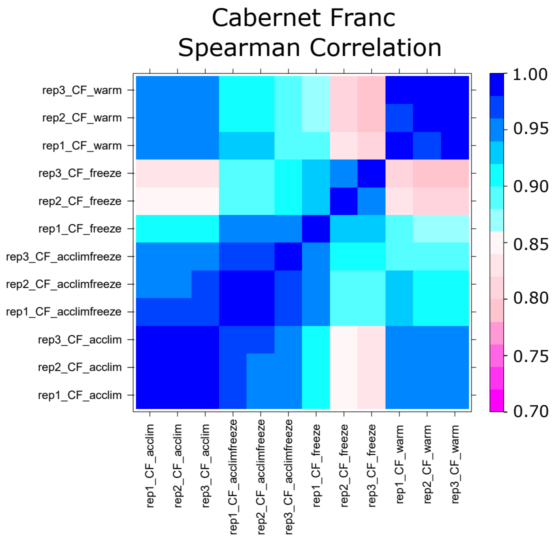
Cabernet Franc
Spearman Correlation
rep3_CF_warm
rep2_CF_warm
rep1_CF_warm
rep3_CF_freeze
rep2_CF_freeze
rep1_CF_freeze
rep3_CF_acclimfreeze
rep2_CF_acclimfreeze
rep1_CF_acclimfreeze
rep3_CF_acclim
rep2_CF_acclim
rep1_CF_acclim
rep1_CF_acclim
rep2_CF_acclim
rep3_CF_acclim
rep1_CF_acclimfreeze
rep2_CF_acclimfreeze
rep3_CF_acclimfreeze
rep1_CF_freeze
rep2_CF_freeze
rep3_CF_freeze
rep1_CF_warm
rep2_CF_warm
rep3_CF_warm
1.00
0.95
0.90
0.85
0.80
0.75
0.70

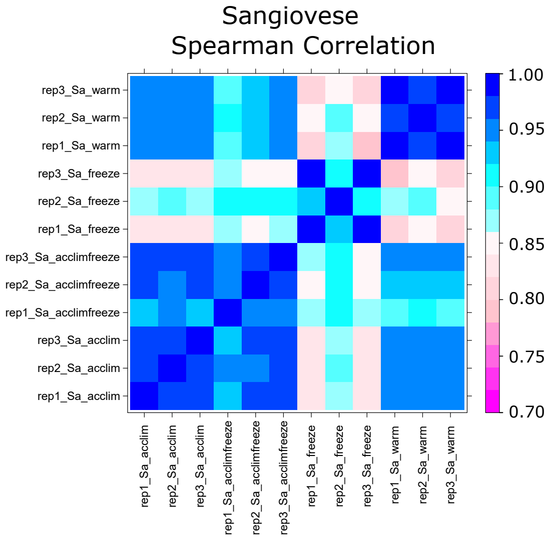
Sangiovese
Spearman Correlation
rep3_Sa_warm
rep2_Sa_warm
rep1_Sa_warm
rep3_Sa_freeze
rep2_Sa_freeze
rep1_Sa_freeze
rep3_Sa_acclimfreeze
rep2_Sa_acclimfreeze
rep1_Sa_acclimfreeze
rep3_Sa_acclim
rep2_Sa_acclim
rep1_Sa_acclim
rep1_Sa_acclim
rep2_Sa_acclim
rep3_Sa_acclim
rep1_Sa_acclimfreeze
rep2_Sa_acclimfreeze
rep3_Sa_acclimfreeze
rep1_Sa_freeze
rep2_Sa_freeze
rep3_Sa_freeze
rep1_Sa_warm
rep2_Sa_warm
rep3_Sa_warm
1.00
0.95
0.90
0.85
0.80
0.75
0.70

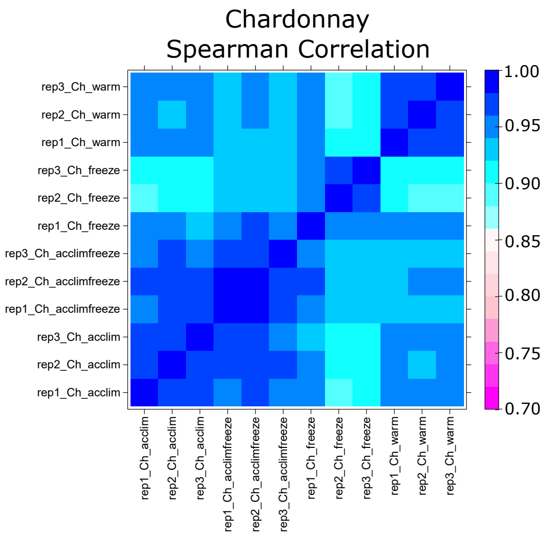
Chardonnay
Spearman Correlation
rep3_Ch_warm
rep2_Ch_warm
rep1_Ch_warm
rep3_Ch_freeze
rep2_Ch_freeze
rep1_Ch_freeze
rep3_Ch_acclimfreeze
rep2_Ch_acclimfreeze
rep1_Ch_acclimfreeze
rep3_Ch_acclim
rep2_Ch_acclim
rep1_Ch_acclim
rep1_Ch_acclim
rep2_Ch_acclim
rep3_Ch_acclim
rep1_Ch_acclimfreeze
rep2_Ch_acclimfreeze
rep3_Ch_acclimfreeze
rep1_Ch_freeze
rep2_Ch_freeze
rep3_Ch_freeze
rep1_Ch_warm
rep2_Ch_warm
rep3_Ch_warm
1.00
0.95
0.90
0.85
0.80
0.75
0.70

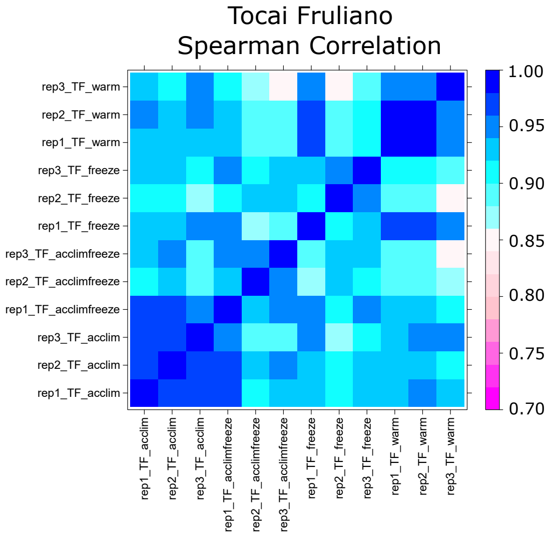
Tocai Fruliano
Spearman Correlation
rep3_TF_warm
rep2_TF_warm
rep1_TF_warm
rep3_TF_freeze
rep2_TF_freeze
rep1_TF_freeze
rep3_TF_acclimfreeze
rep2_TF_acclimfreeze
rep1_TF_acclimfreeze
rep3_TF_acclim
rep2_TF_acclim
rep1_TF_acclim
rep1_TF_acclim
rep2_TF_acclim
rep3_TF_acclim
rep1_TF_acclimfreeze
rep2_TF_acclimfreeze
rep3_TF_acclimfreeze
rep1_TF_freeze
rep2_TF_freeze
rep3_TF_freeze
rep1_TF_warm
rep2_TF_warm
rep3_TF_warm
1.00
0.95
0.90
0.85
0.80
0.75
0.70

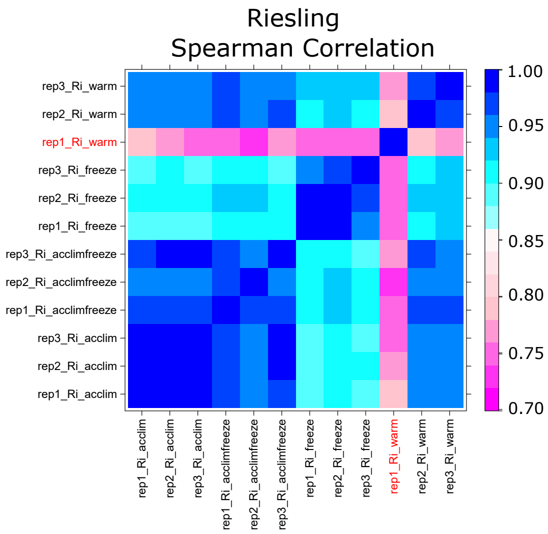
Riesling
Spearman Correlation
rep3_Ri_warm
rep2_Ri_warm
rep1_Ri_warm
rep3_Ri_freeze
rep2_Ri_freeze
rep1_Ri_freeze
rep3_Ri_acclimfreeze
rep2_Ri_acclimfreeze
rep1_Ri_acclimfreeze
rep3_Ri_acclim
rep2_Ri_acclim
rep1_Ri_acclim
rep1_Ri_acclim
rep2_Ri_acclim
rep3_Ri_acclim
rep1_Ri_acclimfreeze
rep2_Ri_acclimfreeze
rep3_Ri_acclimfreeze
rep1_Ri_freeze
rep2_Ri_freeze
rep3_Ri_freeze
rep1_Ri_warm
rep2_Ri_warm
rep3_Ri_warm
1.00
0.95
0.90
0.85
0.80
0.75
0.70
