# Supplementary figures and images for: Divergence in the transcriptional landscape between low temperature and freeze shock in cultivated grapevine (Vitis vinifera)
Source: Hortic Res. 2018 Mar 1;5:10. doi: 10.1038/s41438-018-0020-7 (PMC5830407; doi:10.1038/s41438-018-0020-7)

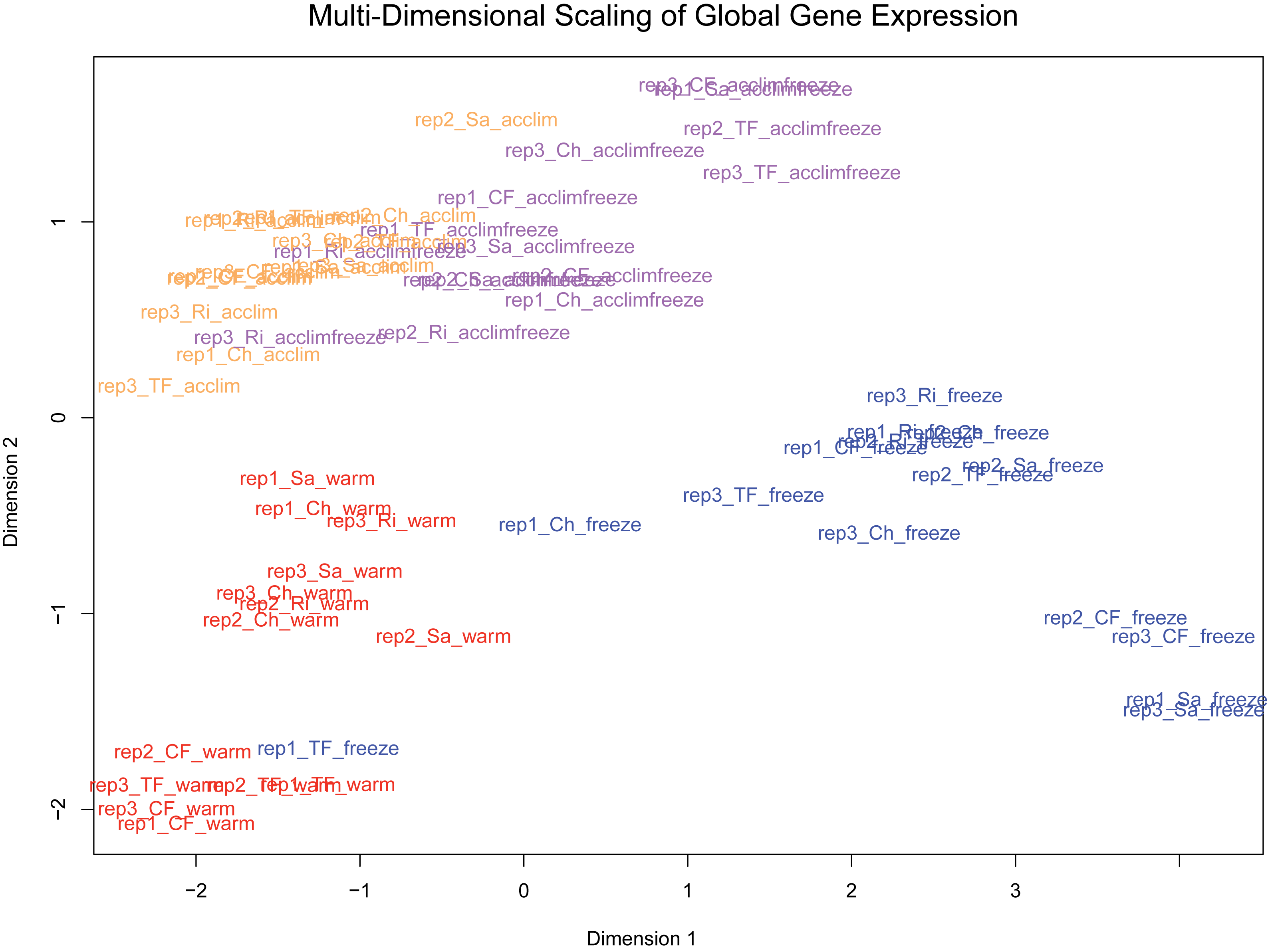

Supplement: Supplementary file 2 — Supplemental Figure 2 [file 41438_2018_20_MOESM2_ESM.tif]

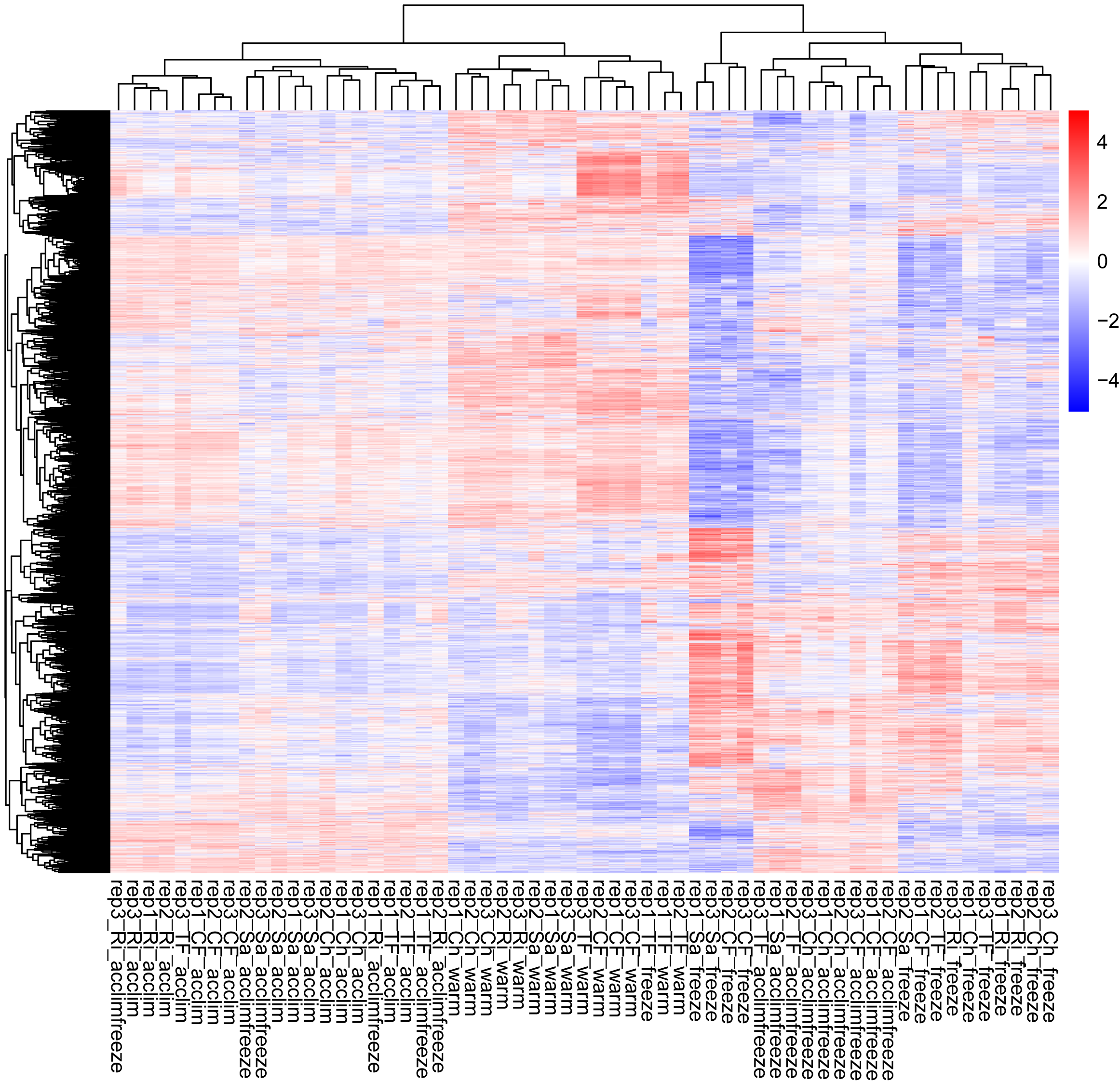

Supplement: Supplementary file 3 — Supplemental Figure 3 [file 41438_2018_20_MOESM3_ESM.tif]
